# Prenatal Fetal Neurocutaneous Melanosis: A Case Report and Literature Review

**DOI:** 10.1002/mgg3.70174

**Published:** 2025-12-31

**Authors:** Xue Zhao, Jing Wang, Juan Song, Yiwen He, Xiaoying Zhang, Qiang Ma, Ying Gu, Li Lin

**Affiliations:** ^1^ Department of Obstetrics and Gynecology Peking University International Hospital Beijing China; ^2^ Department of Pathology Peking University International Hospital Beijing China

**Keywords:** case report, cerebellar hemispheres, neurocutaneous melanosis, prenatal diagnosis

## Abstract

**Background:**

Neurocutaneous melanosis (NCM) is a rare congenital syndrome characterized by congenital melanocytic nevus of the skin with melanocytic deposits in the central nervous system. Patients with neurological symptoms have a poor prognosis and may die within years of symptom onset.

**Case Report:**

A fetus was found to have diffuse enhancement of cerebellar hemispheric echo at 23 weeks of gestation and amniocentesis was performed at 25 weeks of gestation. Fetal DNA was extracted from amniotic fluid for copy number variation sequencing (CNV‐seq) and Trio‐total whole‐exome sequencing (Trio‐WES). However, genetic tests did not reveal pathogenic mutations associated with this case phenotype. At 29 weeks of pregnancy, a fetoscopy examination was performed, and multiple scattered pigmentation spots were found on the skin of the fetus's back. At 31 weeks of pregnancy, the pregnant woman requested an induced abortion to terminate the pregnancy. Multiple areas of pigmentation can be seen on the skin of a stillborn fetus. Pathological examination confirmed a large amount of melanin deposition in the cerebellum tissue of the stillborn fetus.

**Conclusions:**

We reported a rare case of prenatal NCM, but no known pathogenic mutations, such as *NRAS* gene mutations, were found. This confirmed that there might be no definite pathogenic mutations in the NCM case, providing important data support for the prenatal identification and diagnosis of NCM.

We reported a rare case of prenatal NCM, but no known pathogenic mutations, such as NARS gene mutations, were found. This confirmed that there might be no definite pathogenic mutations in the NCM case, providing important data support for the prenatal identification and diagnosis of NCM.

## Graphical abstract

A multimodal prenatal diagnostic approach for Neurocutaneous Melanosis (NCM), integrating ultrasound, MRI, fetoscopy, and genetic testing, in a case with negative NRAS/BRAF mutations.

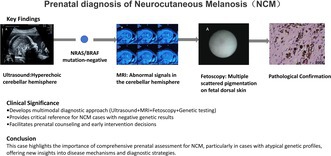

## 1 Introduction

Neurocutaneous melanoma (NCM) is a rare congenital syndrome caused by neuroectodermal dysplasia and characterized by congenital melanocytic nevus of the skin and melanocytic deposits in the central nervous system. There are more than 100 clinical cases reported, of which pediatric cases account for the majority, but prenatal cases are still rare. We report a rare case of prenatal NCM detected by ultrasound, MRI, and fetoscopy during pregnancy and confirmed pathologically. Genetic testing in this case did not reveal any known associated pathogenic mutations, a finding that is important for prenatal identification and diagnosis of NCM.

## 2 Case Report

**FIGURE 1:**
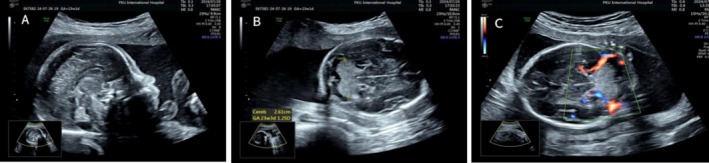
Fetal color Doppler ultrasound at 23 weeks of gestation. (A) Grayscale ultrasound of the fetal posterior fossa, showing bilateral symmetric diffuse hyperechogenicity of cerebellar hemispheres; vermis intact, no intracranial abnormalities. (B) Axial grayscale ultrasound of the fetal posterior fossa, demonstrating symmetric diffuse hyperechogenicity of cerebellar hemispheres. (C) Color Doppler ultrasound of the fetal cerebellum, revealing diffuse hyperechogenicity of cerebellar hemisphere with normal arterial and venous blood flow.

**FIGURE 2:**
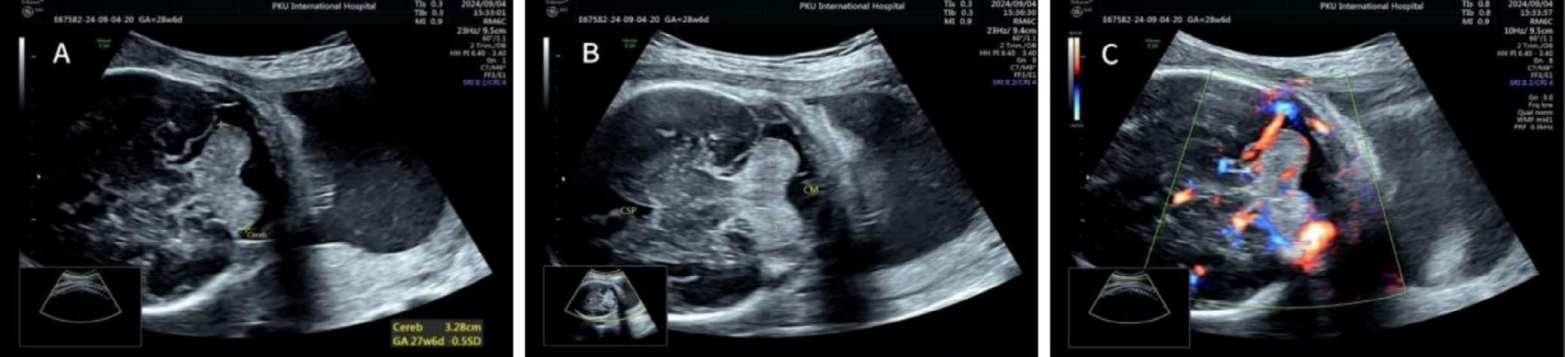
Fetal color Doppler ultrasound at 28^+6^ weeks of gestation. (A) Grayscale ultrasound of the fetal posterior fossa, presenting persistent bilateral symmetric diffuse hyperechogenicity of cerebellar hemispheres (slightly reduced intensity vs. 23 weeks); vermis intact. (B) Axial grayscale ultrasound of the fetal posterior fossa, showing diffuse hyperechogenicity of cerebellar hemispheres (slightly nonuniform parenchyma); adjacent structures normal, no hydrocephalus. (C) Color Doppler ultrasound of the fetal cerebellum, demonstrating diffuse hyperechogenicity of cerebellar hemisphere with regular blood flow perfusion.

**FIGURE 3:**
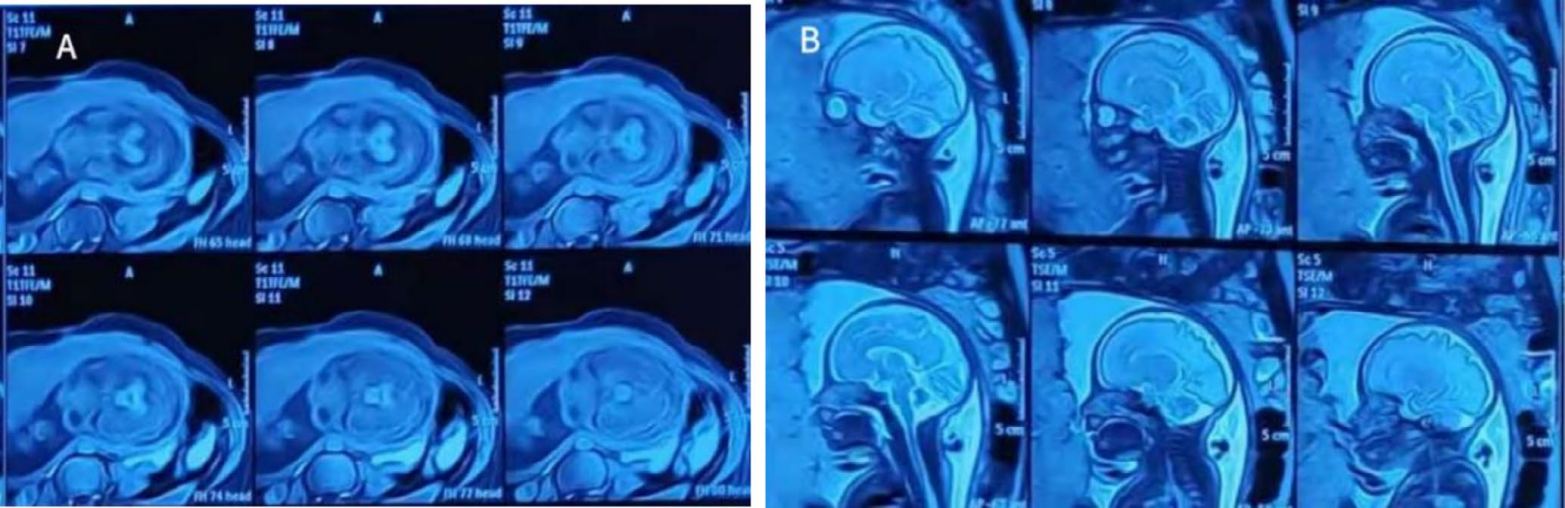
Fetal brain MRI at 29^+5^ weeks of gestation (T1‐weighted [T1WI] and T2‐weighted [T2WI] sequences). T1WI (A): Focal hyperintense signals in the bilateral cerebellar hemispheres, consistent with potential melanin deposition. T2WI (B): Corresponding hypointense signals in the same cerebellar regions, correlating with T1WI findings and supporting suspicion for melanosis (or hemorrhage/excessive myelination).

**FIGURE 4:**
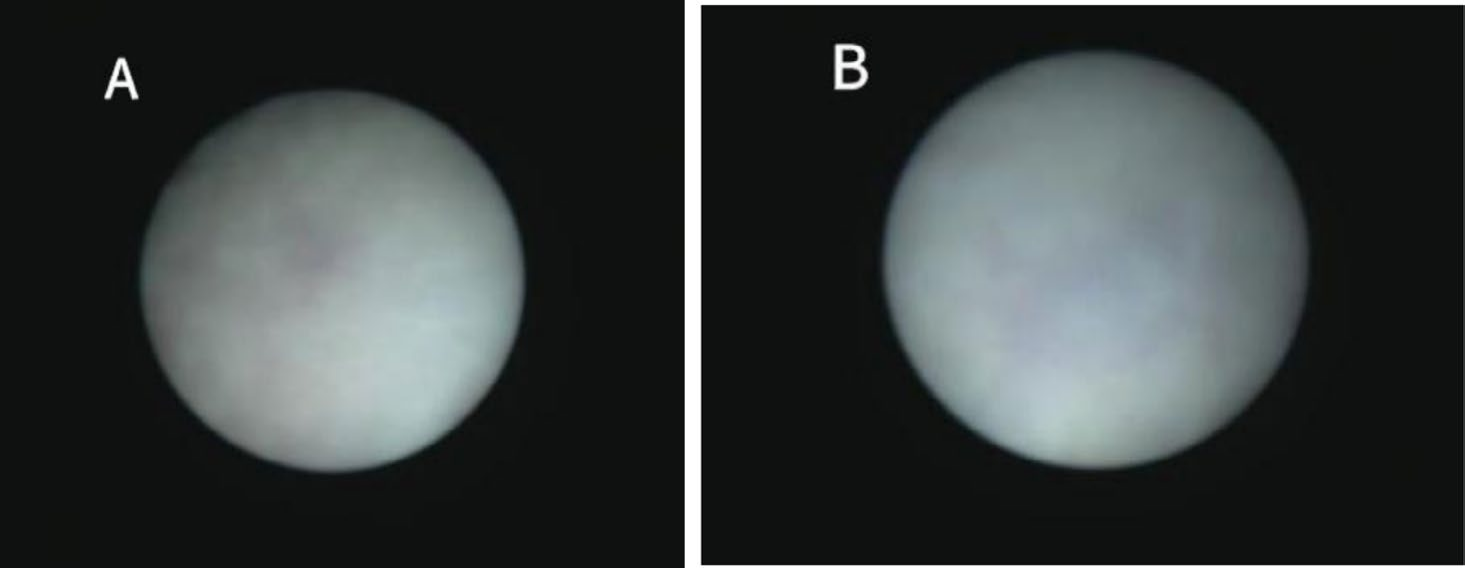
Fetoscopy images of the fetal dorsal skin. (A, B) Direct intrauterine visualization of the fetal back showing multiple scattered, small pigmented macules (brownish‐black, non‐confluent lesions) on the dorsal cutaneous surface. No ulceration, elevation, or large congenital melanocytic nevi are noted.

In this case, the pregnant woman was 38 years old, G4P2A1, with no history of adverse pregnancy, delivered twice vaginally, and had two healthy children. There was no abnormality in fetal ultrasound at 13 gestational weeks, with NT 1.5 mm. Fetal color Doppler ultrasound examination at 23 weeks of gestation (Figure 1) shows normal cerebellar hemisphere size, visible vermis, and diffuse enhancement of cerebellar hemisphere echo. Fetal brain MRI at 24 weeks of gestation showed no definite abnormality of fetal nervous system. In the blood of the pregnant woman, TORCH detection indicated increased CMV‐IgM. The pregnant woman subsequently underwent amniocentesis at 25 weeks and 6 days of pregnancy, during which CNV‐Seq, Trio‐WES, and tests for CMV‐DNA and parvovirus B19 were conducted on the amniotic fluid. CMV‐DNA in amniotic fluid was negative, with < 4.00E + 2 copies/mL, and parvovirus B19 was negative. CNV‐Seq detection of amniotic fluid showed 0.62 Mb duplication of X chromosome, which was considered a benign mutation. The pathogenic mutations linked to the phenotype of this instance were not found by Trio‐WES testing. Fetal color Doppler ultrasound examination conducted at 28 weeks and 6 days of gestation (Figure 2) showed diffuse enhancement of fetal cerebellar hemisphere echo, slightly lower echo intensity than before, less uniform cerebellar parenchyma echo than before, with no obvious abnormality found in other intracranial structures. Fetal brain MRI at 29 weeks of gestation (Figure 3) showed abnormal signals in the cerebellar hemisphere of the fetus, suggesting possible melanosis, hemorrhage, or excessive myelination. Subsequently, the pregnant woman underwent fetoscopy (Figure 4). The procedure revealed the fetal dorsal region, demonstrating multiple scattered pigmented areas on the skin; however, no biopsy was performed. At 31 weeks of pregnancy, the patient consulted at the genetic counseling clinic of our hospital, and were informed that the fetus had cerebellar lesions and was likely to have cerebellar ataxia and language disorder after birth. After thoughtful reflection, both the wife and her husband chose to terminate the pregnancy and relinquish the fetus. After reporting to the ethics committee of the hospital, the pregnant woman was admitted to the ward for induction of labor.

**FIGURE 5:**
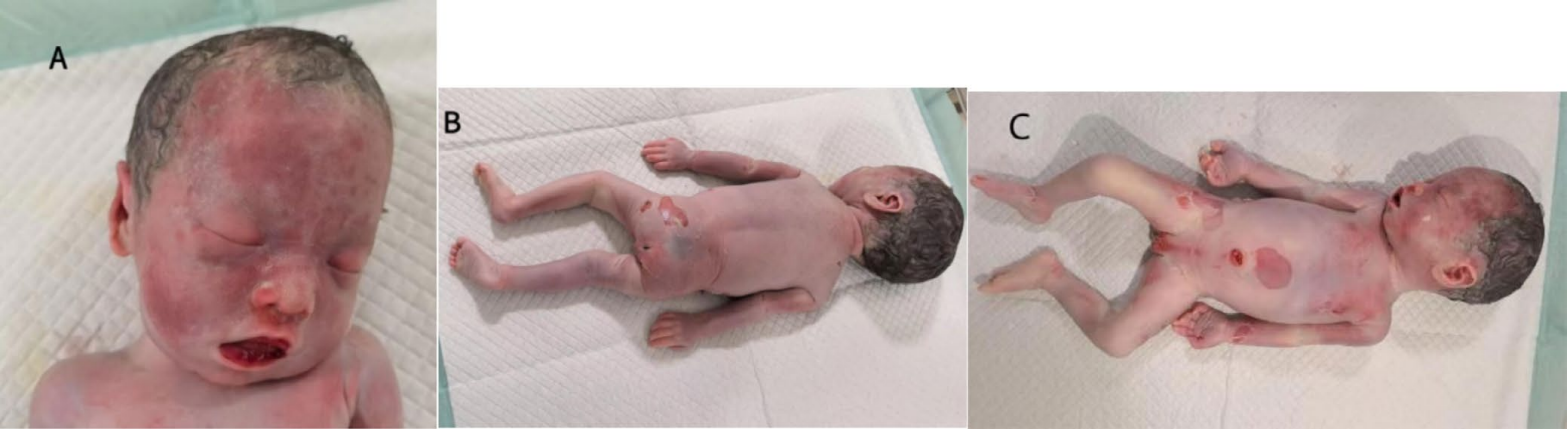
Cutaneous melanosis of the stillborn fetus. (A) Frontal‐mandibular region: Diffuse patchy brown melanosis extending from the frontal bone to the mandible. (B) Sacrococcygeal region: A single well‐circumscribed melanotic lesion, measuring approximately 2.5 × 2.5 cm, with uniform pigmentation; no satellite lesions are seen. (C) Anterior full‐body gross view: Gross morphology of the stillborn fetus showing no additional overt melanotic lesions or structural anomalies on the anterior torso and extremities.

**FIGURE 6:**
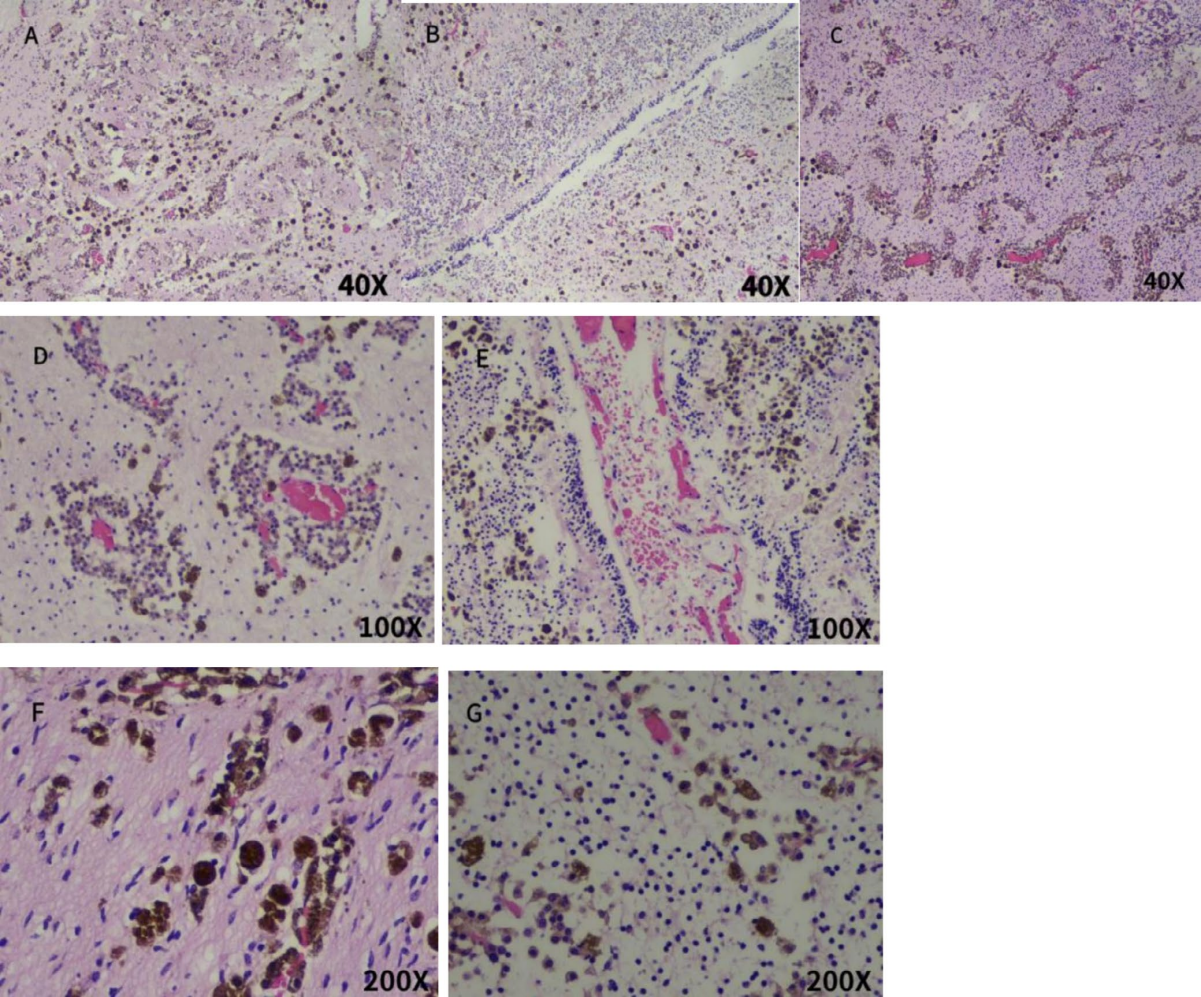
Microscopic examination of the stillborn fetus's cerebellar tissue (Fontana–Masson staining). (A–C) ×40 magnification: (A) Melanin in cerebellar molecular layer; (B) melanin in granular layer; (C) panoramic view of widespread brown granular melanin infiltration. (D, E) ×100 magnification: (D) Perivascular melanin aggregation; (E) focal parenchymal melanin deposition. (F, G) ×200 magnification: (F) Virchow–Robin spaces filled with non‐heteromorphic round melanocytes; (G) melanocyte detail (no mitoses, necrosis, or neural invasion).

A dead female infant was discharged through vagina on the second day after induction of labor, which weighed 1820 g and was 45 cm long. The skin of frontal bone to the mandible of the dead infant showed multiple patchy melanosis, and one spot of melanosis was seen on sacrococcyx skin, covering an area of about 2.5 × 2.5 cm (Figure 5). The appearance of other parts was not obviously abnormal. The cerebellum of the deceased infant was collected for pathological analysis. Microscopically, large amounts of melanin were deposited in the cerebellar parenchyma and perivascular Virchow‐Robin spaces (Figure 6). The cells were round and did not appear to be heteromorphic. At the microscopic level, there were no signs of hemorrhage, necrosis, or invasion of nerves. However, no considerable melanin was detected in the pia mater. In the end, the pregnant woman elected against proceeding with a systematic autopsy of the stillborn fetus. Based on the clinical manifestations of melanosis in the skin of the dead infant, combined with the pathological findings of cerebellum tissue, this case was diagnosed as NCM.

Six months after the induction of labor, the woman had another spontaneous pregnancy. The prenatal examination was uneventful throughout the pregnancy. Amniocentesis was refused for prenatal diagnosis in the second trimester. Fetal malformation screening ultrasound at 22 and 28 weeks of gestation showed no abnormality. A live baby girl was born by spontaneous labor at 36 weeks of gestation. All indicators of the newborn were good, and no congenital cutaneous melanocytic nevus or neurological abnormalities were found by the pediatrician.

## 3 Discussion

NCM is a rare congenital syndrome, first reported by Bohemian pathologist Rokitansky in 1861, with an incidence of approximately 1 in 20,000 to 50,000 (Ruggieri et al. 2020; Islam 2015). The clinical features of NCM are primarily evident in two systems. NCM presents on the one hand as congenital melanocytic nevus (CMN) of the skin, proliferative melanocytic nodules (PNs), or malignant transformation of previous melanotic lesions into melanomas. CMN can be a single giant mole (LCMN) or multiple moles (MCMN) or both, or it can be accompanied by satellite moles (Ruggieri et al. 2020; Kadonaga and Frieden 1991). On the other hand, NCM is characterized by melanocyte deposition (with infiltration) in the brain parenchyma or pia mater, which leads to neurological symptoms. Melanin can be visualized on MRI T1‐weighted imaging and appears as solid nodular or linear T1 hyperintense lesions in the pia mater, especially in the myelinated brain (Islam 2015; Gocmen et al. 2015).

Typically, NCM skin melanotic lesions are recognized at birth. Neurological symptoms typically manifest within the first 2 years after birth, primarily due to progressive hydrocephalus resulting from leptomeningeal melanosis infiltration. These symptoms may include intracranial hypertension, seizures, cranial nerve dysfunction, developmental delay, and, in rare cases, Dandy‐Walker syndrome, arachnoid cysts, or spina bifida occulta (Tercanli et al. 2016; Sarwar et al. 2021). In some cases, children exhibit prenatal central nervous system (CNS) manifestations, as seen in our case. Children with neurological symptoms generally have a poor prognosis, as most of them died within 3 years of symptom onset (Islam 2015; Ji et al. 2025). Around 40%–60% of symptomatic patients are at risk of developing melanoma, which can deteriorate their prognosis. A rare case of adult neurocutaneous melanosis combined with intracranial primary malignant melanoma was reported in 2018 (Ma et al. 2018). The patient was born with a giant congenital nevus and satellite nevus on the skin. At the age of 34 years, the patient was diagnosed with primary intracranial malignant melanoma. The patient was treated with surgery but forwent further radiotherapy and chemotherapy. Six months after surgery, the patient's MRI showed recurrence of intracranial melanoma, but she refused another operation and died 22 months after surgery. The authors recommend that children with giant congenital melanocytic nevi undergo regular comprehensive examinations, with particular focus on the central nervous system (e.g., via brain and spinal cord MRI) to rule out neurocutaneous melanosis. Once identified, early aggressive treatment should be initiated to improve prognosis.

The incidence of NCM is fairly low, and there are currently more than 100 cases of NCM reported, most of which are concentrated in children or individual adults during adolescence, but prenatal detection of NCM cases is very rare. In one case, macrocephaly, ventricular ectasia, and posterior fossa effusion were detected by ultrasound at 34 weeks of gestation. Further fetal MRI revealed a large posterior fossa cyst, resulting in ventricular enlargement. The newborn was discovered to have melanocytic nevi at the time of birth. Brain MRI on day 3 also showed significant melanin deposition in the cerebellum and amygdala (East and Soares 2021). Another patient was found to have a solitary cyst measuring 16 × 33 × 23 mm in the fetal posterior fossa, detected by ultrasound at 28 weeks of gestation and confirmed as an arachnoid cyst through fetal MRI. Cysts did not show any tendency to increase in size during pregnancy. After birth, the newborn was found to have giant melanotic nevus with satellite phenomenon. T1‐weighted sequences of neonatal MRI revealed substantial melanosis in the brain, typically located in the amygdala (unilateral), pons, thalamus, and frontal lobe (Tercanli et al. 2016).

In 1991, Kadonaga and Frieden (1991) summarized the diagnostic criteria for NCM: (1) large nevus (> 20 cm in adults and lesions which are approximately 9 cm of diameter on the head or 6 cm on the body in infants), (2) multiple (≥ 3) nevi, (3) no evidence of cutaneous melanoma, except in cases where meningeal lesions are histologically benign, (4) no evidence of meningeal melanoma, except in cases where the cutaneous lesions are benign. Typical skin lesions can be observed at birth, while neurological symptoms typically emerge afterward. Two‐thirds of moles are made up of a large axial congenital melanocytic mole, which may or may not have satellite moles, while the other one‐third of the various moles predominantly appear on the trunk or head. The diagnosis of neurological disorders depends on physical examination, ultrasound, brain or spinal MRI, EEG, intelligence tests, etc. MRI might reveal meningeal thickening surrounding the brain and spinal cord, with characteristic high signal intensity on T1‐weighted sequences and low signal intensity on T2‐weighted sequences, indicating the presence of melanin and suggesting a diagnosis of NCM. Brain melanosis is a common presentation in children with cutaneous melanocytic nevi. It occurs most commonly in the anterior temporal lobe (amygdala), brain stem, cerebellum, and cerebral cortex. Early imaging provides the highest diagnostic sensitivity (Jakchairoongruang et al. 2018). Diagnosis relies on microscopic examination of pathological samples, particularly those from the nervous system. In the central nervous system, the affected pericerebral space is filled with melanocytes. These cells are not heteromorphic, mitotic, necrotic, or hemorrhagic. They invade the Virchow‐Robin space around blood vessels, but do not invade neural tissue. Virchow‐Robin space is a typical feature of NCM diagnosis because melanocytes are not normally present at these locations (Kadonaga and Frieden 1991; Caldarelli et al. 2005). The clinical or imaging diagnosis of NCM can be checked by genetic testing for *NRAS* gene mutations.

According to current research, NCM is thought to be caused by postzygotic mutations at codon 61 of the *NRAS* gene, which causes *NRAS* mutation mosaicism (Kinsler et al. 2013; Charbel et al. 2014). *NRAS* is a homologous oncogene of the neuroblastoma RAS virus, situated on chromosome 1p13.2. The signal of the *NRAS* protein product is passed on to the nucleus, which can indicate cell division or proliferation. Mutations in the *NRAS* protooncogene disrupt *RAS/MEK/ERK* and *PI3K‐Akt* pathways, driving melanocyte proliferation in utero, leading to single/multiple CMNs and central nervous system lesions (Charbel et al. 2014; Abqari et al. 2016). Some researchers have suggested that NCM can also be caused by somatic mutations in the *BRAFV600E* gene (Ichii‐Nakato et al. 2006; Salgado et al. 2015). One study (Salgado et al. 2015) indicated that 75% of the NCM patients analyzed had *NRASQ61* mutations (specifically *NRASQ61K* and *NRASQ61R*), while 12.5% of NCM patients had the *BRAFV600E* mutation, and another 12.5% showed no presence of either *BRAFV600* or *NRASQ61* mutations. Point mutations or gene fusions in other genes (e.g., mutations in *BRAF, KRAS, APC*, and *MET* genes, or gene fusions in *ZEB2‐ALK* and *SOX5‐RAF1* genes) may be associated with melanoma proliferation or melanoma progression in satellite nevi or LCMN (Martins Da Silva et al. 2019).

To date, the majority of NCM cases reported in the literature have been found to have somatic mutations at codon 61 of *NRAS*. However, there are a lot of NCM instances with no known pathogenic mutations. Kinsler et al. (2013) reported a case of NCM that lacked any *NRASQ61* mutations and did not provide further details on other potentially pathogenic mutations. Another case report (Uguen et al. 2015) described two examples of prenatal onset of severe fetal hydrocephalus caused by NCM. Case 1 reported the same *NRASQ61K* mutation in cutaneous melanotic nevus and meningeal hyperplasia. However, case 2 did not find any known related mutations. Trio‐WES also did not diagnose any pathogenic gene mutations associated with NCM in our case. Therefore, further research is needed to explore other factors or molecular mechanisms that influence NCM and thus explain the melanin deposition in brain tissue and pia mater.

Skin melanosis and nervous system infiltration of NCM are usually benign, and a few may progress to melanoma or nonmalignant melanosis of the brain. However, if neurological symptoms occur, the prognosis is usually poor and may even lead to fetal death, adolescent or adult death. Current treatments for NCM include laser therapy, chemical peeling, dermabrasion, and surgical removal of CMN, as well as surgical removal/chemotherapy/radiotherapy of neurological lesions (Ruggieri et al. 2020). However, the current treatment methods are basically to improve neurological symptoms, and there is still no established treatment method that can cure NCM. Targeted therapy presents a promising treatment avenue, and while MEK inhibitors have been documented for addressing the symptomatic NCM attributed to the *NRASQ61K* mutation, it is noted that delayed therapy did not avert mortality (Küsters‐Vandevelde et al. 2014; Sarkisian and Davar 2018). Nonetheless, there are histological indications of reduced cell proliferation linked to this targeted therapy. Another in vitro cell study (Basu et al. 2016) found that inhibitors of the *NRAS* signaling pathway specific mediators reduced survival of *NRAS* mutant cells, with the best effect obtained with *GSK2126458*, showing a reduction in mutant cell survival of less than 50%, which confirms that drugs targeting the *NRAS* signaling pathway reduced the viability of NCM cells in vitro. These data imply that the *NRAS* signaling pathway is crucial to the future targeting of NCM.

This article reports a case of NCM with typical skin manifestations and corresponding neuropathological diagnosis discovered prenatally. However, no known pathogenic gene mutation was found. It provides important data support for prenatal identification and diagnosis of NCM. More research in this field is needed to further clarify the pathogenesis of NCM and explore more targeted therapies.

## Author Contributions

Xue Zhao: conceptualization, formal analysis, writing – original draft. Jing Wang: supervision, validation, writing – review and editing. Juan Song: data curation – collect and organize clinical medical record data. Yiwen He: formal analysis – provide guidance in maternal‐fetal medicine. Xiaoying Zhang: formal analysis – provide guidance in pathological diagnosis. Qiang Ma: formal analysis – provide guidance on prenatal diagnostic ultrasound. Ying Gu: formal analysis – provide guidance in genetic prenatal diagnosis. Li Lin: supervision, validation.

## Funding

This study was funded by the project entitled “Preclinical Study on the Treatment of Intrauterine Adhesions with Placental Mesenchymal Stem Cells.”

## Consent

We have obtained written informed consent from the patient to publish details of her medical case and any accompanying pictures of herself and her fetus.

## Data Availability

The data that support the findings of this study are available from the corresponding author upon reasonable request.
